# Qualification of proxy experiments for accelerated electrochemical testing in self-driving labs

**DOI:** 10.1007/s00216-026-06609-9

**Published:** 2026-06-19

**Authors:** Annica Heyne, Mert Ozan, Ahmad Rashid Hazem, Ozlem Ozcan

**Affiliations:** Federal Institute for Materials Research and Testing (BAM), Unter Den Eichen 87, 12205 Berlin, Germany

**Keywords:** Material acceleration platforms, MAPs, Self-driving labs, Corrosion, Electrochemical analysis

## Abstract

Material acceleration platforms (MAPs) aim at accelerating the discovery of functional materials by combining simulation-assisted pre-screening of potential candidate materials with fast property evaluation using self-driving labs (SDLs) enabling an early upscaling and assessment of device level properties. For the development of corrosion protection technologies or in energy applications, a key challenge lies in identifying property descriptors that reliably predict long-term stability while remaining compatible with accelerated workflows. Conventional corrosion tests for long-term performance remain indispensable but include procedures that can last from hours to months which renders them unsuitable for early-stage screening within large chemical and microstructural design spaces. Consequently, there is a critical need for time‑efficient electrochemical measurements that retain predictive value to determine candidate materials and can be seamlessly integrated into automated platforms. This study presents a comparative evaluation of the corrosion properties of four different alloys (CrCoNi, FeCrNi, CrMnFeCoNi, and AISI 304) in 3.5 wt% NaCl at pH 7.0, with the objective of developing a minimal yet informative electrochemical sequence suitable for SDL deployment. We outline a systematic approach to designing electrochemical proxy experiments: first, by identifying the corrosion processes most relevant to the target application; second, by assessing candidate techniques with respect to their diagnostic value relative to experimental time; and finally, by refining the sequence based on data interpretability and operational flexibility. For the investigated alloys and conditions, chronopotentiometry was the most effective method both in terms of the property differentiation and execution time.

**Graphical Abstract:**

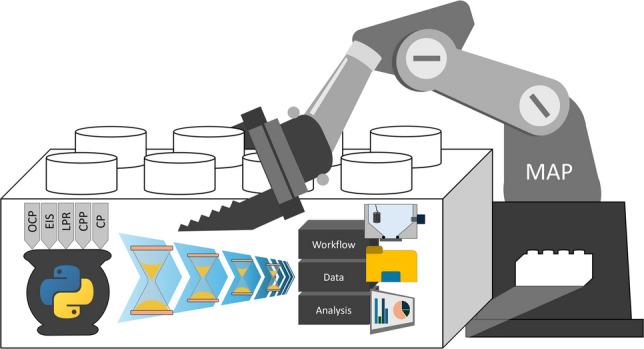

## Introduction

Societal challenges, including climate change, rising energy demand, and rapid consumption of critical resources, are accelerating the need to discover advanced materials on compressed timelines. With the rise of robotic lab integration, process automation, optimization, and analysis through machine learning (ML) and artificial intelligence (AI), a paradigm shift in advanced materials discovery and evaluation has taken place and catalyzed the development of material accelerating platforms (MAPs) [1, 2]. In fully operational configurations, MAPs leverage self-driving laboratories (SDLs) to autonomously plan and execute experiments, use simulation-assisted pre-screening to prioritize viable candidates, and apply ML to interpret results. The MAP approach offers fast property evaluation enabling faster innovation cycles through early upscaling and assessment of application relevant properties.

Among the societal challenges, corrosion imposes substantial economic cost by degrading structural and functional performance across engineering systems and technologically intricate components. This motivates the search for corrosion-resistant alloys (CRAs) with superior and tunable properties. High-entropy alloys (HEAs) or multi-principal element alloys (MPEAs) have emerged as promising candidates combining desirable properties such as excellent corrosion behavior with outstanding mechanical properties [3–6]. By comprising multiple principal elements, MPEAs open vast, previously underexplored compositional spaces [7, 8], and their compositional complexity has yielded alloys with unexpected properties, including enhanced corrosion resistance [9–11].

Conventional corrosion evaluation typically involves electrochemical testing complemented by surface-specific or even in situ analyses to gain mechanistic insight [12–15]. These campaigns are time- and labor-intensive, and without computational pre-selection, identifying CRAs becomes even slower. Recent data-centric advances begin to address this bottleneck. Nyby et al. [16] compiled a CRA dataset (including MPEAs) spanning eight corrosion parameters extracted from 85 publications across four material classes, facilitating ML-guided corrosion prediction and alloy design. Song et al. [17] developed cross-modal knowledge graphs to capture processing, composition, and experimental diversity in the HEA Corrosion Dataset. This dataset underpins their corrosion prediction with structural prediction (CPSP) framework [18], which models the interdependence of composition, processing, crystal structure, and corrosion behavior. While CPSP currently focuses on structured learning and predicting corrosion resistance via ln(*j*_corr_) (logarithm of corrosion current density) in 3.5 wt% NaCl, it exemplifies the value of integrating and evolving computational pre-screening within MAPs.

Complementary MAP advances target high-throughput simulation, fabrication, and testing. Zeng et al. [19] demonstrate a simulation-based ML framework that enables the high-throughput prediction of corrosion parameters relying on key metrics such as single-phase formability, surface energy, and Pilling-Bedworth ratio. Using CALPHAD, DFT, and surrogate models, Antokainen et al. [20]. demonstrated systematic, high-throughput HEA fabrication and characterization with SOLID-MAP. On the corrosion testing side, Sur et al. [21] introduced a scanning droplet cell for high-throughput electrochemical evaluation of MPEAs, extracting corrosion potential (*E*_corr_), passive (*j*_pass_) and critical current densities (*j*_crit_), and the impedance modulus |Z| at 0.01 Hz from open circuit potential (OCP), linear sweep voltammetry (LSV), and electrochemical impedance spectroscopy (EIS), respectively, on magnetron-sputtered Al_0.7−x_Co_x_Cr_y_Fe_0.15_Ni_0.15_ libraries. In related work, high-throughput screening of magnetron-sputtered Al-, Co-, and Cr-rich MPEAs enabled a funnel from broad screening (e.g., XRD-identified single-phase BCC MPEAs) to high-fidelity electrochemical analysis [1, 2].

A persistent challenge is identifying high-throughput electrochemical metrics that are sensitive to localized corrosion (e.g., pitting), which is harder to detect, often autocatalytic, and less predictable than uniform corrosion [22, 23]. Although polarization-derived parameters predominantly reflect uniform corrosion [16], practical screening requires metrics—or combinations thereof—that also reveal localized attack. Even widely used pre-measurement OCP holds (approx. 10 min–1 h) [24–26] may not stabilize on such timescales [27], complicating protocol standardization and leading to erroneous data. Here, we assess the information content of electrochemical parameters from open-circuit potential (OCP), electrochemical impedance spectroscopy (EIS), linear polarization resistance (LPR), and cyclic potentiodynamic polarization (CPP) to propose a minimal yet insightful electrochemical sequence for corrosion resistance determination for future SDL application. We further evaluate chronopotentiometry (CP) previously used to study pit nucleation and repassivation potentials [28–31], as a time‑efficient alternative to lengthy CPP scans for detecting localized corrosion.

**Fig. 1:**
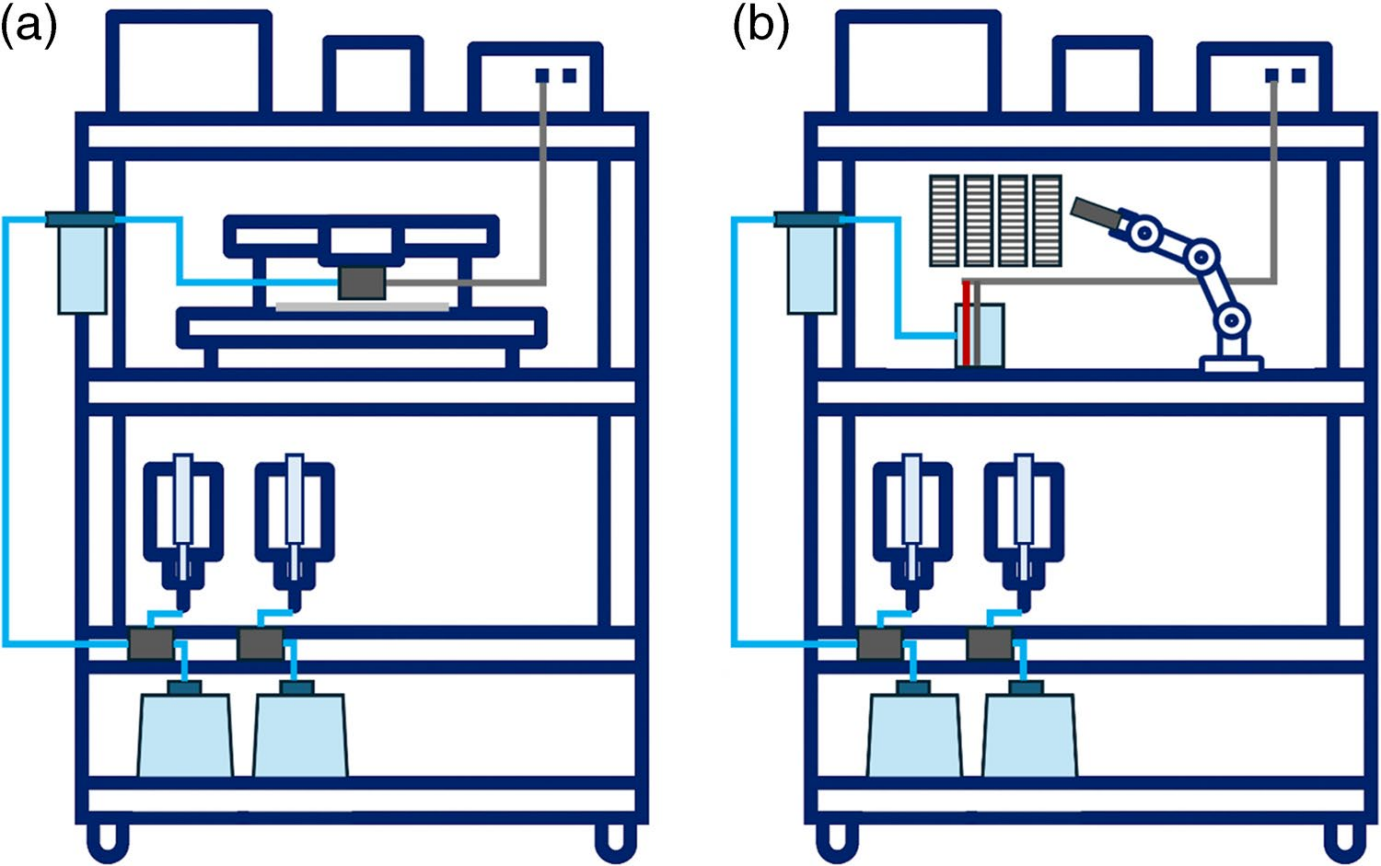
The experimental sequences are designed for deployment in two different MAP set-ups that share a common liquid handling module. The two systems differ in their sample handling approach: In (**a**), measurements are performed using a scanning electrochemical cell that seals a defined area on a large sample sheet. In (**b**), individual samples are arranged in a rack and picked up by a robotic arm using a gripper. In this configuration, electrochemical tests are carried out in a funnel beaker, which enables replacement of the electrolyte between runs

We evaluated four alloys to identify corrosion parameters that robustly rank their resistance in 3.5 wt% NaCl at neutral pH as a condition chosen for literature comparability and to avoid confounding factors (pH, temperature). Two MPEAs, CrCoNi and FeCrNi, serve as corrosion-resistant benchmarks [32], while CrMnFeCoNi and AISI 304 represent pitting-susceptible systems in chloride electrolytes [33]. All experiments are scripted in Python to streamline future integration into autonomous MAP workflows (see Fig. 1).

## Material and methods

### Materials

Four different alloys were investigated in this study. For the three representative samples of the MPEA class, equi-molar CrMnFeCoNi, CrCoNi, and FeCrNi were used. All vacuum-melted MPEA ingots were manufactured by the Laplanche group of Ruhr-University, Bochum, Germany. To briefly summarize, after casting, the CrMnFeCoNi and CrCoNi alloys were homogenized and recrystallized for 1 h at 1020 °C to 1060 °C, respectively. Both MPEAs exhibited a single-phase fcc microstructure with a mean 50 µm grain size for CrMnFeCoNi and 16 µm for CrCoNi [34, 35]. Similarly, FeCrNi was homogenized and recrystallized for 2 h at 1200 °C. It exhibited a 95 vol% fcc microstructure and 5 vol% bcc-structure microstructure that precipitated at the grain boundaries. The grain size was at 8 µm [36]. Table 1 displays the chemical compositions of all three MPEAs and was taken as reported [34, 36]. Herein, the full descriptions on the alloy preparation and microstructural characteristics can also be found.

**Table 1:** Chemical composition of CrCoNi, FeCrNi, and CrMnFeCoNi in atomic % as reported [34, 36]

	Co	Cr	Fe	Mn	Ni	C	O	S
CrCoNi	33.30	32.53	0.95	0.09	32.85	0.019	0.226	0.004
FeCrNi	-	33.78	32.15	-	33.89	0.032	0.125	0.003
CrMnFeCoNi	19.70	19.41	20.56	20.10	19.58	0.051	0.033	0.009

Fully annealed austenitic stainless-steel sheets (AISI 304, Goodfellow Cambridge Ltd., UK) were obtained as 1.2-mm-thick sheets and cut into coupons of 1.5 × 1.5 cm dimensions. The chemical composition of AISI 304 is summarized in Table 2 as reported [37].

**Table 2:** Weight percentages (wt%) of elements present in the AISI 304 alloy apart from Fe, detected by inductively coupled plasma optical emission spectrometry [37]

	Mn	Cr	Ni	C	S, N, Si, P
AISI 304	0.97	18.52	8.02	0.035	≪ 1

### Sample preparation

All alloy specimens were wet ground from 240 to 600 grit SiC paper. Then they were rinsed in an ultrasonic bath (Elmasonic P, Germany) in deionized water (0.055 µS cm^−1^, Evoqua, USA), followed by ethanol (ChemSolute, min. 99.5%, Germany) for 5 min each at 80 kHz to remove any residue on the surfaces. Afterwards the specimens were dried in an oil-free stream of compressed air. Finally, the specimens were transferred into a drying oven at 120 °C to force passive film formation. The samples were removed and stored in a desiccator under vacuum until the final transferal into the electrochemical cells.

### Electrochemical measurements

All electrochemical experiments in sequence were conducted through a Python script with Emstat4S HR™ potentiostats (PalmSens BV, Netherlands). The electrochemical cells consisted of a three-electrode set-up with a Ag/AgCl 3M NaCl (ALS, Japan) reference electrode, a gold auxiliary electrode, and a sample alloy as the working electrode. The measurements were conducted in 3.5 wt% NaCl (Chemsolute, 99.0%; Germany, in deionized water (0.055 µS cm^−1^, Evoqua, USA)) at pH 7, at room temperature with no extra aeration strategy. To run the experiment sequence of OCP_1_-EIS_1Hz_-OCP_2_-EIS_0.1Hz_-OCP_3_-EIS_0.01Hz_-OCP_4_-LPR-OCP_5_-CPP the respective MethodSCRIPT™ for each experiment was extracted from the PSTrace software (PalmSens BV, version 5.11). Employing the Python script examples provided by PalmsSens on their respective GitHub repository, Python scripts were written to control all experiments through a main Python script orchestrating the different sequences of the full electrochemical experiment run and enabling to run 4 cells at the same time.

OCP_1–3_ were conducted for 5 h; OCP_4_ and OCP_5_ were run for 60 s. Impedance spectra were run from 100,000 Hz to different low frequencies ranging from 1 to 0.01 Hz. LPR measurements were run within a ± 0.015 V potential range from OCP at a scan rate of 0.1 mV/s. CPP scans were run from −0.25 V vs. OCP to an apex potential of 1.5 V vs. OCP at 0.1 mV s^−1^. On top of the sequence, freshly prepared specimens were investigated through chronopotentiometry by stepwise application of increasing current densities of 2.5, 5, 10, and 25 µA cm^−2^ and monitoring the measured potential over 300 s for each current density with Emstat4S LR™ potentiostats (PalmSens BV, Netherlands). The measurement sequence has been run three times on each freshly prepared alloy to ensure the reproducibility of the data. For all alloys, the first measurement was chosen as the representative dataset. The collected data for each sequence were converted to csv files to be readable by Python and exported to data analyzing and graphing tools such as origin. The full code and data for the experimental part as well as the data analysis segments can be found on Zenodo [38].

## Results and discussion

To assess which electrochemical measurements and corresponding corrosion parameters are suitable to be integrated as part of an SDL in a MAP, a sequence of typical electrochemical measurements was run in 3.5 wt% NaCl. This electrolyte was purposefully chosen, first to have a highly corrosive environment where surface changes due to corrosion are anticipated and second to allow for data comparability as many corrosion studies in the literature consider this electrolyte. Chloride-containing electrolytes are highly aggressive towards an alloy’s passive oxide film and may cause its breakdown through local disintegration resulting in pitting [39]. In previous studies, pitting corrosion in various NaCl electrolytes has been observed for AISI 304 and equi-molar CrMnFeCoNi [33, 40]. For equi-molar CrCoNi, research has indicated local corrosion in the form of intergranular corrosion [32]. While Fe-18Cr-14Ni (wt%) succumbs to pitting corrosion [13], for the equi-molar FeCrNi MPEA, no local corrosion mechanism has been identified to date. With four channels available in this proxy experimentation set-up, all four alloys were investigated simultaneously in one run. The proxy experimental sequence comprised the following experiments: OCP_1_-EIS_1Hz_-OCP_2_-EIS_0.1Hz_-OCP_3_-EIS_0.01Hz_-OCP_4_-LPR-OCP_5_-CPP. From the final experiment (CPP), the passivation current could be withdrawn and was used to run a separate chronopotentiometry measurement, to investigate passive film stability. In general, the procedure begins with the application of non‑destructive electrochemical techniques—OCP, EIS, and LPR—and concludes the CPP measurement, which intentionally perturbs the surface through imposed anodic corrosion. The intermediate OCP measurements are included to assess the stability of the alloys’ passive film. In a separate measurement, CP was employed to determine its applicability for localized corrosion detection.

The first experiment in the sequence comprises OCP monitoring. Here, no current or potential is applied to the electrochemical system; only the potential difference between the working electrode (alloy sample) and the reference electrode is monitored over time. The OCP corresponds to the potential of an electrochemical system at which the anodic and cathodic reaction rates equal each other [16]. At this point, no net current flows through the system and changes to the OCP are due to surface reactions such as oxide formation, metal dissolution, or hydrogen/oxygen reduction, i.e., corrosion. Such surface reactions may change the interface chemistry locally due to concentration changes in near-surface dissolved ions which in turn will have an effect on the overall surface potential and show as change in OCP. The OCP is a typical electrochemical parameter in corrosion studies [41]. While for measurements such as CPP or EIS, it serves as a point of reference, and *E*_OCP_ is often cited and interpreted as a corrosion parameter in the literature [16, 42]. Typically, the OCP is recorded before a measurement to assure that the system reaches a steady state whereas timeframes for collection comprise 10 to 60 min [24–26]. Sensitive to ongoing surface reactions and environmental factors, such as temperature, humidity, oxygen saturation of the electrolyte, or pre-treatment and storage of the interface, the OCP can vary and give different values for the same system. Consequently, interpreting the OCP in isolation—without careful assessment and complementary analytical methods—is inherently challenging. In practice, the temporal evolution of the potential is typically used to evaluate a system’s stability within a given electrolyte. To monitor how reliable the OCP is as a corrosion parameter, we included it in the proxy experiment sequence to screen its stability over a longer time.

**Fig. 2:**
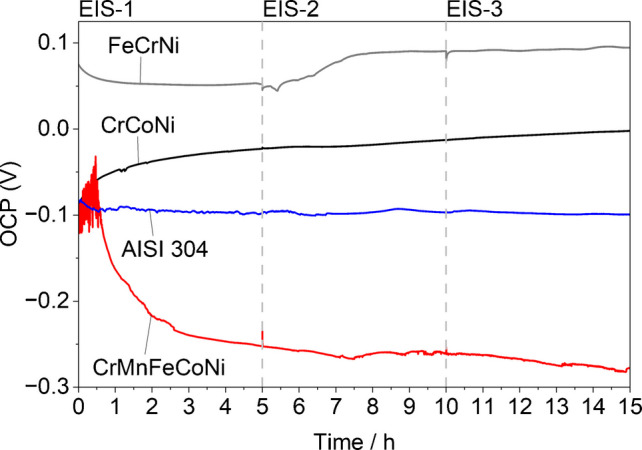
Consecutive open circuit potentials for the indicated alloys recorded for 5 h before EIS-1, 2, and 3

To deduce the feasibility of the OCP as a corrosion parameter in a MAP optimization loop, it was recorded prior to each analysis and collected for 5 h. We allowed for this long timeframe due to the proxy set-up to monitor the interface stability. Figure 2 illustrates the perpetual development of the OCP for each alloy over a 15-h period. Each 5-h OCP measurement was taken before acquiring an EIS to observe whether a stable state could be reached within an extended timeframe. The most notable instability in OCP was observed for CrMnFeCoNi, which exhibited strong fluctuations within the first hour spanning a potential range of approximately 0.6 V. Both ternary MPEAs showed an increase in OCP over time; however, CrCoNi displayed a much more stable and linear progression. For FeCrNi, a more pronounced increase in OCP occurred after 5 h, flattening out at around 7.5 h. AISI 304 exhibited fluctuations as well, although less severely than CrMnFeCoNi, with noticeable more abrupt changes until approximately 7 h, after which only slight variations persisted over time.

**Fig. 3:**
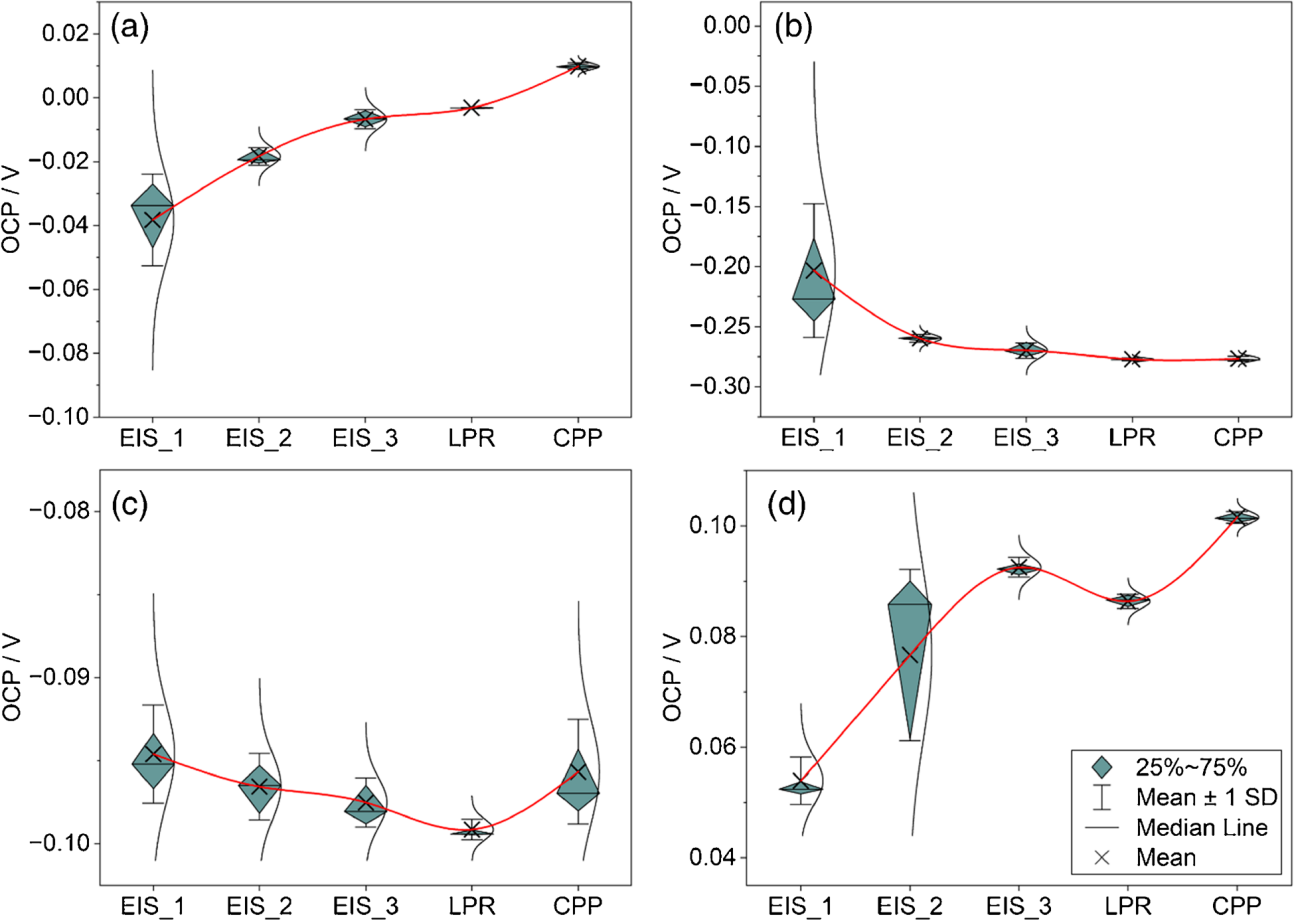
OCP development over the whole experimental sequence for (**a**) CrCoNi, (**b)** CrMnFeCrNi, (**c)** AISI 304, and (**d**) FeCrNi. Each box graph represents the OCP data collected before each experimental sequence and the OCP development is depicted by connection of the mean of each set. The diamond box graphs reflect the interquartile range, and the standard deviation is depicted by the whiskers

While Fig. 2 illustrates the direct development of the OCPs measured before each EIS, Fig. 3 summarizes all collected OCP data, including the two measurements recorded prior to the LPR and CPP scans. The graphs in Fig. 3a–d present the evolution of OCPs over time taken before the respective electrochemical measurement. In these graphs, the OCP is shown as the mean value from each measurement with its respective interquartile range and standard deviation. For both CrCoNi and FeCrNi, the mean OCP exhibits a linear increase until the OCP measurements before LPR and CPP, whereas for CrCoNi the steepness of the slope decreases, and for FeCrNi its OCP decreases before the LPR and increases again before the CPP. In contrast, the OCP decreases for CrMnFeCoNi and AISI 304, although the steel’s OCP shows a slight increase again before the final measurement. During the initial 5-h OCP collection period, most alloys exhibit relatively high standard deviations in their OCP values. Despite this variability, a consistent shift in the mean OCP value is observed across all samples, as highlighted by the connecting lines in Fig. 3. This behavior underscores the transient nature of the OCP and its sensitivity to ongoing electrochemical processes at the material surface.

**Fig. 4:**
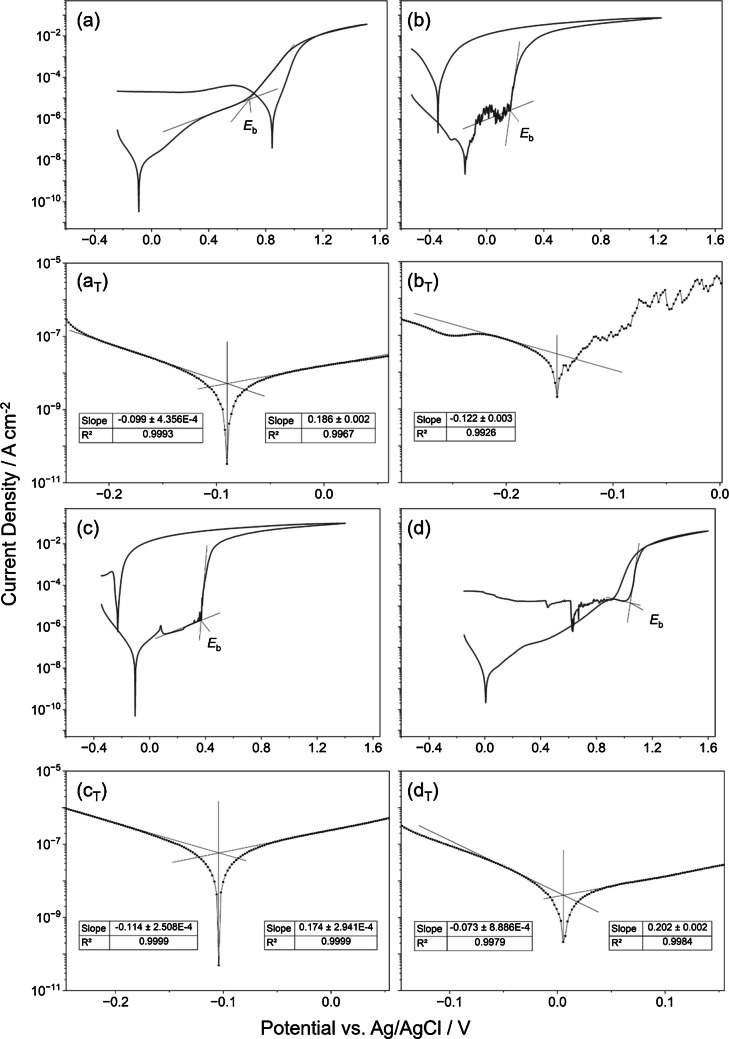
CPP diagrams of (**a**) CrCoNi, (**b)** CrMnFeCrNi, (**c)** AISI 304, and (**d)** FeCrNi, and their respective Tafel regions *a*_T_–*d*_T_. Each CPP graph shows the breakdown potential *E*_b_. Within the Tafel regions, the anodic and cathodic branch fits are indicated whereas the respective slopes and *R*^2^ are presented in the inset tables

Generally, the OCP reflects the potential at which the rates of anodic and cathodic reactions are balanced. Importantly, OCP does not represent a thermodynamic equilibrium as the anodic and cathodic reactions are not equally reversible. This is evidenced by its continual shift over time for all alloys. This lack of stability is further supported by the asymmetric Tafel slopes observed in Fig. 4.The biggest advantage of the OCP is the ease of collection, nondestructive nature, and no necessity for further complicated data processing which makes it a viable parameter to collect. In an SDL electrochemical analysis station, it is easy to integrate OCP between steps while keeping the collection times relatively short.

Although CPP is the final electrochemical measurement in the sequence, it is beneficial to examine its insights prior to analyzing the EIS and LPR data. CPP scans provide a rich dataset from which various corrosion-related parameters can be derived. These parameters include corrosion potential (*E*_corr_), corrosion current density (*j*_corr_), passive potential range, breakdown potential (*E*_b_), transpassive region, repassivation potential (*E*_rp_), and indicators of localized corrosion. The *E*_corr_ and *j*_corr_ can both be extracted from fitting the cathodic and anodic branch of the scan within the Tafel region of the CPP data as shown in Fig. 4. This region corresponds to the portion of the potential scan where the measured current transitions from negative to positive values as the potential increases. In Fig. 4, the insets show the respective Tafel regions and fits for each alloy. Table 3 summarizes key corrosion parameters derived from the CPP scans. Among the tested alloys, FeCrNi exhibits the lowest *j*_corr_, followed by CrCoNi, while AISI 304 shows the highest. Notably, the *j*_corr_ for AISI 304 and CrMnFeCoNi are approximately one order of magnitude higher than those of CrCoNi and FeCrNi which in turn translates to higher corrosion rates [43]. While *j*_corr_ is a general corrosion parameter and a common metric in corrosion databases, the extracted values must be interpreted with caution. In this study, a slow scan rate of 0.1 mV s⁻^1^, a 2-s equilibration period between data points, and a highly concentrated electrolyte were employed to minimize IR-drop effects [44]. Nevertheless, capacitive charging contributions to the measured current cannot be fully ruled out. Prior work by Zhang et al. shows that *E*_corr_ obtained from low scan rate potentiodynamic measurements during anodic polarization aligns closely with *E*_OCP_, although the corresponding *j*_corr_ and anodic Tafel slope may be higher [45]. Moreover, decreasing the scan rate is known to increase the interfacial capacitance, which can further influence the measured response.

**Table 3:** Summary of electrochemical parameters derived from CPP of (a) CrCoNi, (b) CrMnFeCrNi, (c) AISI 304, and (d) FeCrNi

	*j*_corr_ [nA cm^−2^]	*E*_corr_ [V]	*b*_a_ [V dec^−1^]	*b*_c_ [V dec^−1^]	*E*_b_ [V]
a	8.73 ± 4.00	−0.16 ± 0.07	0.16 ± 0.02	−0.11 ± 0.01	0.75 ± 0.12
b	39.66 ± 19.23	−0.17 ± 0.02	-^a^	−0.11 ± 0.01	0.15 ± 0.03
c	45.23 ± 12.93	−0.06 ± 0.03	0.17^b^	−0.11 ± 0.02	0.37 ± 0.03
d	5.13 ± 1.12	0.08 ± 0.05	0.22 ± 0.03	−0.08 ± 0	0.99 ± 0.03

^a^Anodic branch could not be fitted

^b^Anodic branch could not be fitted for all measurements

At potentials above the Tafel region (shifted to more positive potentials), CrCoNi and FeCrNi exhibit a somewhat stable passive state in the 3.5 wt% NaCl electrolyte. While for a perfect passive region, the currents would remain stable over a certain potential range, and the measured currents slightly increase, indicating active growth or a not fully protective passive layer [46, 47]. In contrast, AISI 304 and CrMnFeCoNi show current fluctuations beyond the Tafel region during the forward scan, suggesting the presence of localized corrosion [48]. The positive hysteresis further confirms the occurrence of pitting corrosion. Interestingly, CrMnFeCoNi demonstrates metastable pit formation and re-passivation even within the potential range of the OCP, indicating early onset of localized corrosion. This early onset of pitting renders the evaluation of the anodic branch problematic for this alloy. Consequently, Tafel analysis becomes ineffective, as it assumes uniform corrosion behavior, which is not present in this case. As discussed in McCafferty’s work [43], when pitting corrosion disrupts the linearity of the anodic branch, only the cathodic branch should be used for deducing corrosion currents. For CrMnFeCoNi, this approach was consistently necessary and, in some instances, also for AISI 304. Both alloys demonstrate susceptibility to pitting corrosion, which complicates standard electrochemical analysis and necessitates alternative evaluation strategies. This is especially important when implementing the CPP for corrosion analysis in a closed-loop, as it is important for the MAP to be able to discard samples that exhibit such detrimental corrosion properties.

Another notable observation is that the breakdown potential which generally marks the transition from the passive to the transpassive region appears to be a characteristic and reproducible parameter across the different alloy systems studied, even at this high corrosive load. For alloys that succumb to pitting corrosion, *E*_b_ also corresponds to the pit nucleation potential [28]. The breakdown potential (*E*_b_) remained consistent across all measurements, with a standard deviation of 0.03 V for CrMnFeCoNi, AISI 304, and FeCrNi. CrCoNi showed a slightly higher standard deviation of 0.12 V. But *E*_b_ may change with different scanning rates which is important when creating databases [28]. In general, the parameters for recording potentiodynamic data are vital and should be included in corrosion databases to allow for direct comparability. While OCP values tend to shift over time, after the 15 h of stabilization within the scope of this experiment, the measured *E*_corr_ during CPP remained relatively stable for all four alloys. However, CrCoNi and FeCrNi exhibited higher standard deviations in *E*_corr_ compared to the others.

These findings suggest that the combination of *E*_corr_ and *E*_b_ could serve as reliable parameters for assessing the reproducibility of corrosion data in the SDL. While *E*_corr_ alone may not be a dependable indicator of corrosion resistance, it can be effectively used in conjunction with *E*_b_ during optimization runs to validate data consistency. Important to note is that within a single extended OCP measurement run, the OCP value can deviate significantly over time from its initial reading (see Figs. 2 and 3), further emphasizing the need for complementary parameters in corrosion analysis.

While the CPP provides a vast pool of corrosion information and parameters, after a full run, the sample surface has changed due to forced passivation and corrosion during the scan. This renders further electrochemical experiments ineffective afterwards. Moreover, a typical scan can last up to 1 h which in a high-throughput SDL environment is rather undesirable. These drawbacks require either strategic placement of CPP measurement to the end of an electrochemical analysis or to take the measurement apart. Taking it apart could mean that only the Tafel region gets measured within the electrochemical series. This way property descriptors such as *E*_corr_, *j*_corr_, and the Tafel slope can be monitored to estimate the uniform long-term stability and durability. Promising candidates could then be further investigated employing CPP.

**Fig. 5:**
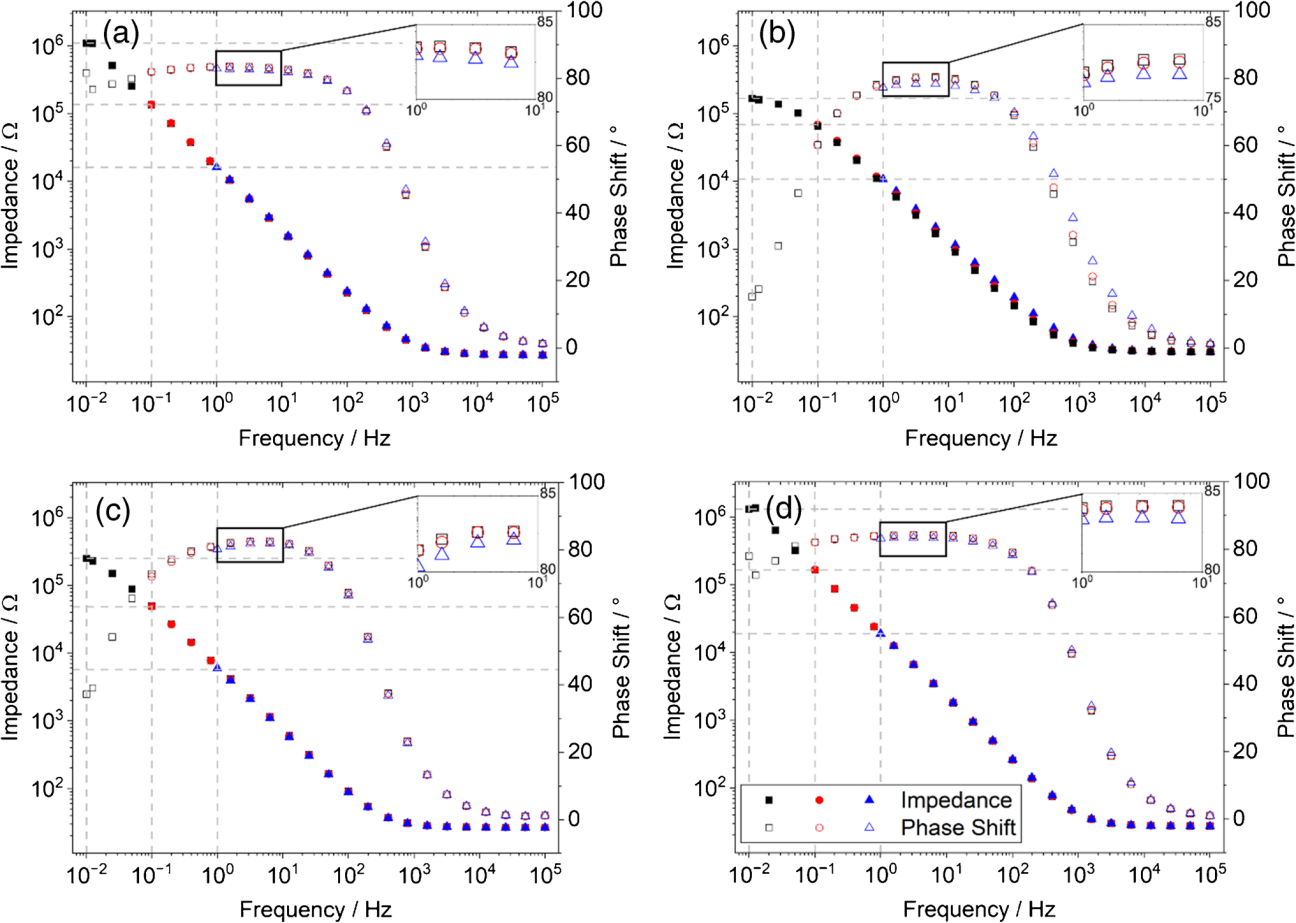
EIS represented as Bode plots of (**a**) CrCoNi, (**b)** CrMnFeCrNi, (**c)** AISI 304, and (**d)** FeCrNi. Each diagram shows the EIS after 1 Hz (blue, triangle), 0.1 Hz (red, circle), and 0.01 Hz (black, square). The impedance moduli at the respective lowest frequency are indicated by the gray dashed lines. The insets in each graph show the phase shifts at medium frequency

The impedance data presented as Bode plots in Fig. 5 display the three impedance measurements recorded for all four alloys. Each measurement was conducted from 100 kHz down to frequencies of 1 Hz, 0.1 Hz, and 0.01 Hz. Reducing the magnitude of the lowest frequency increases the time of the experiment and may lead to alterations in the OCP of unstable alloys if the measurements run for an extended time as demonstrated in Fig. 3. For the 1 Hz, 0.1 Hz, and 0.01 Hz EIS measurements, the expected times surmount to approximately 1 min, 2.5 min, and 17 min, assuming 10 datapoints per decade are collected.

For the effectiveness of the EIS measurement in a MAP context, reliable data is vital. Ideally, fast data collection is chosen over slow data collection to avoid interface changes due to progressive corrosion and to allow high-throughput. In this study, EIS measurements are employed to retrieve |*Z*| at the lowest applied frequency and the maximum phase angle. The impedance spectra were not fitted to a respective electrical equivalent circuit. The comparison between the three measurements serves to determine which frequency limit may be suitable. For CrCoNi, all scans are nearly perfectly aligned, indicating consistent impedance behavior over an extended time which has also been shown in long-term investigations of the MPEA in 1 M H_2_SO_4_ [27]. Both CrCoNi and FeCrNi exhibit broader phase shift curves, suggesting superior passive film stability. However, slight differences in the phase shift are detectable for all alloys over time, where the phase shifts increase marginally (by approximately 0.6–1°) with each consecutive EIS measurement as displayed in the insets of Fig. 5. This phase shift increase suggests general passive film growth and highlights the need to find complementary techniques that can identify localized corrosion apart from CPP [49]. It should be noted that the time difference between the EIS measurements is 5 h which is not a typical timeframe for OCP stabilization. However, despite the noticeable OCP shifts within the corrosive medium over time of immersion, EIS spectra remain reproducible over the timeframe of 15 h. Nevertheless, long-term corrosion studies of CrMnFeCoNi have shown that the MPEA succumbs to severe corrosion in acidic media over long immersion periods [27].

**Fig. 6:**
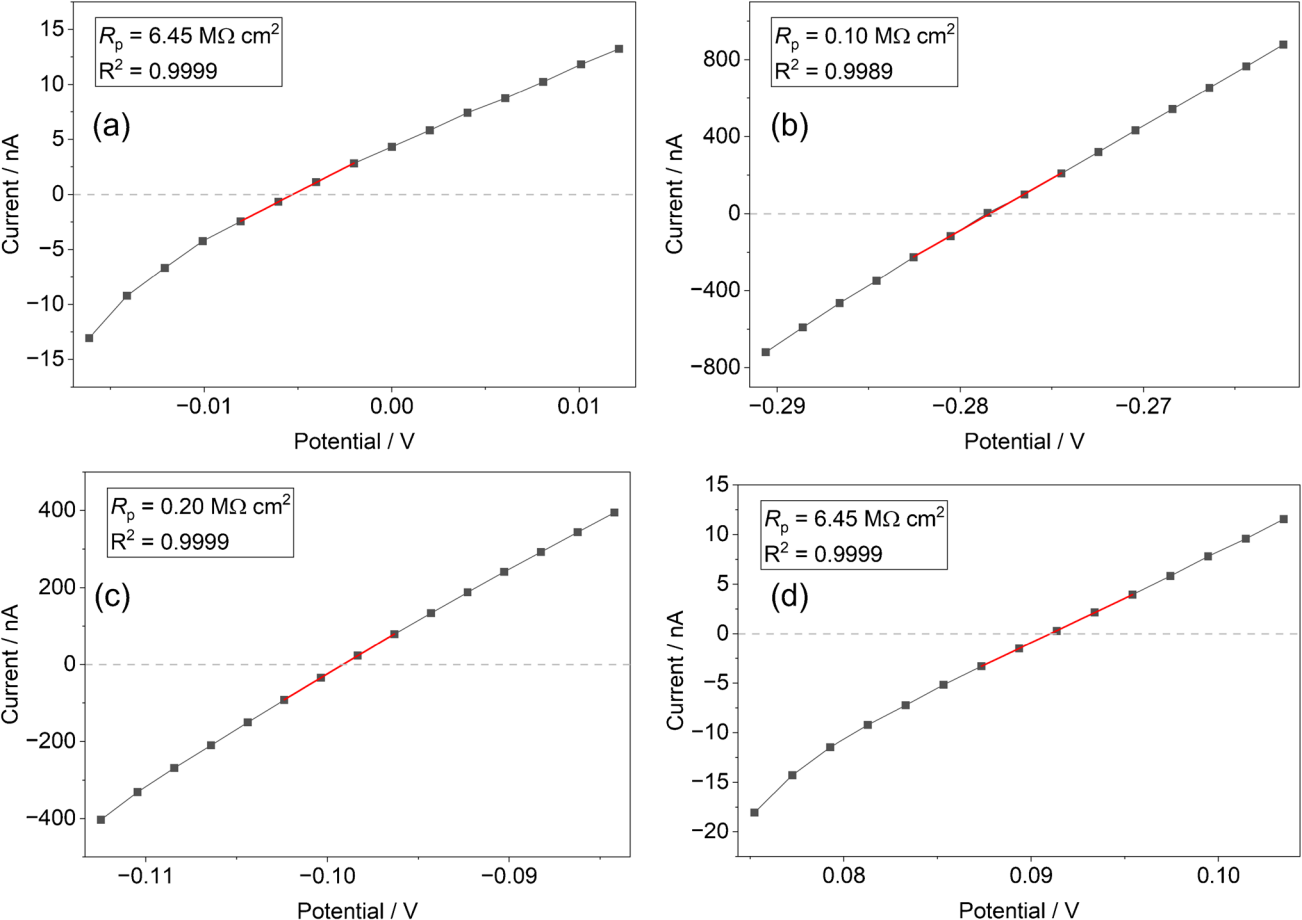
LPR diagrams of (**a**) CrCoNi, (**b)** CrMnFeCrNi, (**c)** AISI 304, and (**d)** FeCrNi. Each diagram shows the linear regression fit for *R*_p_ retrieval through the Python script

In the corrosion literature, the modulus of impedance (|*Z*|) at 0.1 Hz is often stated to correspond to the system’s polarization resistance (*R*_p_) [50]. However, when compared to the *R*_p_ derived from LPR, there are discrepancies between the obtained |*Z*|_0.1Hz_ and *R*_p_ values. Using a potential range of ± 15 mV around the OCP, with a step size of 2 mV and a 2-s equilibration period before each data point, the LPR scan generates 15 data points over approximately 5 min at a scan rate of 0.1 mV/s. In terms of duration, the LPR scan is comparable to an EIS measurement conducted down to 0.1 Hz. Figure 6 shows the LPR graphs and Table 4 summarizes the |*Z*| and *R*_p_ values obtained from the EIS at 0.1 Hz, EIS at 0.01 Hz, and LPR measurements. The linear regression applied to the LPR data was implemented within the Python script, using a fitting window of ± 3 mV, as illustrated by the regression lines in the LPR plots in Fig. 5. The corresponding slopes and associated *R*^2^ values are also reported therein. The |*Z*| from EIS increased from 1 to 0.01 Hz and was closest to the *R*_p_ value determined through LPR at 0.01 Hz. A comparison of the polarization resistances and their respective standard deviations reveals that the LPR-derived *R*_p_ values exhibit significantly greater variability than the |*Z*| values derived from EIS.

**Table 4:** Summary of electrochemical data derived from EIS and LPR of (a) CrCoNi, (b) CrMnFeCrNi, (c) AISI 304, and (d) FeCrNi

	*f*_max_ [°]	*R*_p-LPR_ [MW cm^2^]	*|Z|*_0.01Hz_ [MW cm^2^]	*|Z|*_0.1Hz_ [kW cm^2^]	(DZ/D*f*)_0.01Hz_ [W/Hz]
a	82.73 ± 0.68	3.54 ± 2.83	0.58 ± 0.03	73.58 ± 2.79	0.91 ± 0.01
b	80.90 ± 0.65	0.36 ± 0.18	0.18 ± 0.12	45.18 ± 4.28	0.90 ± 0.01
c	82.53 ± 0.26	3.20 ± 3.17	0.25 ± 0.10	33.98 ± 7.51	0.92 ± 0
d	82.92 ± 1.10	9.94 ± 2.51	0.77 ± 0.04	96.61 ± 3.49	0.92 ± 0.01

Among the tested alloys, FeCrNi shows the lowest deviation between separate LPR measurements, with a relative standard deviation (RSD) of 26.1%. In contrast, AISI 304 displays the highest variability, with an RSD of 99.0%. CrMnFeCoNi and CrCoNi fall in between, with RSDs of 50.7% and 79.9%, respectively. Interestingly, however, the more corrosion-resistant alloys CrCoNi and FeCrNi display a distortion between the *E*_corr_ from LPR and the *E*_OCP_. This can be seen in the LPR diagrams in Fig. 5 where the gray dashed line marks the point of zero current, i.e., *E*_corr_. As discussed for the CPP, this shift may be a result of capacitive charging. In turn, for the EIS-derived |*Z*| values at 0.01 Hz, CrCoNi and FeCrNi demonstrate the most consistent results, with RSDs of 5.0% and 4.8%, respectively. However, CrMnFeCoNi and AISI 304 show much higher variability, with RSDs of 67.8% and 38.5%. The higher variability for CrMnFeCoNi and AISI 304 may be due to the instability of their OCPs over time and pitting corrosion changing the passive film integrity. Interestingly, the RSDs are lowest for all 4 alloys at 0.1 Hz, with 22.1% for AISI 304 being the highest, followed by 9.5% for CrMnFeCoNi, 3.8% for CrCoNi, and 3.6% for FeCrNi.

Additionally, the *R*_p_ and |*Z*| values obtained from LPR and EIS_0.01Hz_ differ by approximately one order of magnitude for both ternary MPEAs. In contrast, for CrMnFeCoNi and AISI 304, the *R*_p_ and |*Z*| values from LPR and EIS_0.01Hz_ are more closely aligned, suggesting a more consistent electrochemical behavior across measurement techniques for these alloys. This may be because if the working electrode in an impedance measurement is ideally polarizable, the transfer of charge between the electrode and the electrolyte takes an infinitesimal rate rendering polarization resistance infinite [51]. This would align with the high *R*_p_ measured through LPR for CrCoNi and FeCrNi to exhibit such high values. The EIS data further shows that we get the most acceptable variation (RSD < 10% for 3 out of 4 alloys) in the |*Z*| at 0.1 Hz which would in turn represent the ideal ranking parameter for corrosion resistance in the MAP.

Following the CPP measurements, the potential range corresponding to the passive region may be identified. After examination of all passive regions, a consistent passivating current density of 2.5 µA cm^−2^ was observed for all four alloys. To investigate the development of the potential starting at a passivating current density, quasi-stationary chronopotentiometry (CP) or galvanostatic polarization was conducted. Each current density was held for 5 min, and potentials were recorded at 2.5, 5, 10, and 25 µA cm^−2^. This was done separately from the measurement sequence since for this proxy experiment passivation currents were discerned first through the CPP. The final applied current density of 25 µA cm^−2^ corresponds to the tranpassive region. Because the CPP changes the alloy surface, the alloy surfaces were freshly prepared for the CP measurements. The chronopotentiometric measurement reflects whether the investigated alloys indicate instabilities due to local passive film breakdown that are large enough to globally cause changes in the measured potential across the entire electrode surface at the applied current densities [31]. Interestingly, CP studies experienced a resurgence in their application in the 60 s and 70 s and Sklarska-Smialowska and Janik-Czachor applied CP to determine pit nucleation and repassivation potentials of Fe-Ni-Cr alloys [28, 30]. According to their research, pits nucleate at *E* ≥ *E*_b_ and will continue to grow below *E*_b_ until they reach *E*_rp_ where they repassivate. Then again, there appears to be a gap in method utilization until the early 2000 s with only a few research studies employing CP. Frangini and De Cristofaro further established that CP can be used to exclude the effect of crevice corrosion on current densities monitored through CPP and extract information on pitting corrosion [29].

**Fig. 7:**
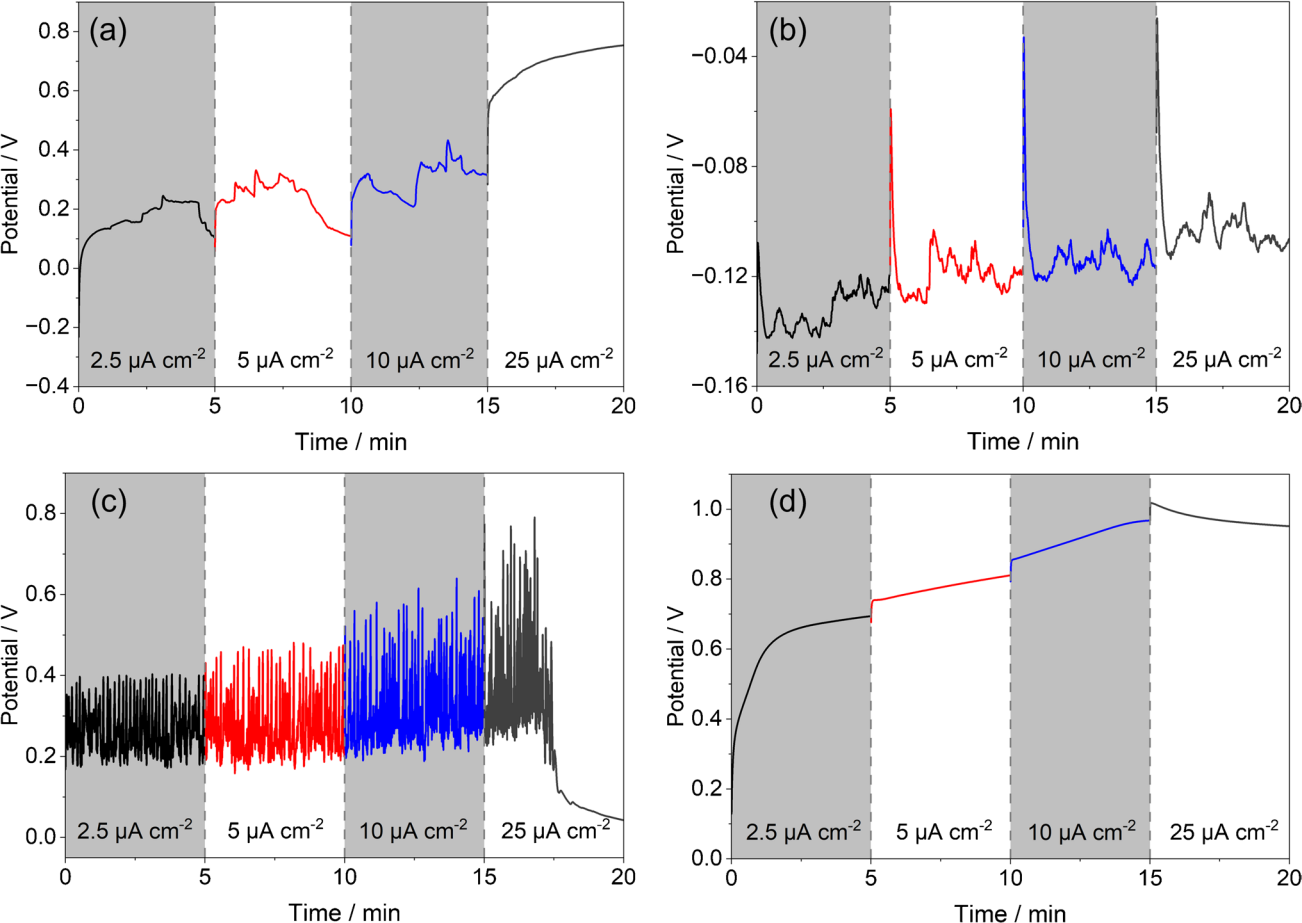
Chronopotentiometry plots of (**a**) CrCoNi, (**b)** CrMnFeCrNi, (**c)** AISI 304, and (**d)** FeCrNi

The results reveal distinct *E-t* curves among the four alloys. CrMnFeCoNi (Fig. 7b) demonstrates continuous potential fluctuations throughout the CP measurement, indicative of the transient nature of pit initiation and repassivation events [28]. The absence of a sustained potential increase suggests that a stable passive film does not form on the MPEA. The sharp rise in potential at the beginning of each current density step corresponds to galvanostatic charging [31]. Generally, it can be observed that the with increasing current density the maximum potential response also increases. Frangini and De Cristofaro argue that the maximum potential recorded in CP reflects the competing processes of pit nucleation and passivation, with high applied current density leading to higher *E*_b_ values [29]. This increase in max measured potential with increasing applied current density can be observed for CrMnFeCoNi, AISI304 and CrCoNi. AISI 304 (Fig. 7c) initially shows an increase in potential with severe oscillations up to approximately 17.5 min, consistent with pit initiation and repassivation [28]. After this point, the potential drops and consistently decreases in a shallow linear fashion whereas no further oscillations are observed and *E*_rp_ is reached. This behavior has been consistently noted in all chronopotentiometry measurements of AISI 304, however the point at which the potential drops varies. Further studies are needed to fully elucidate the surface reactions responsible for these observations. However, research by Tileli and Barbey-Binggeli confirmed the pit nucleation and growth on Al-samples in 0.1 M NaCl through in-operando liquid phase electron microscopy [31]. According to Frangini and De Cristofaro, the potential drop for AISI304 originates from ceasing pit growth over time until it stops under galvonastatic conditions [29].

In contrast, from 2.5 to 10 µA cm^−2^, CrCoNi displays only slight fluctuations in the potential as shown in Fig. 7a. At 25 µA cm^−2^, the potential rises smoothly and seems to stabilize. The measured potential is comparable to the *E*_b_ obtained from CPP. FeCrNi (Fig. 7d) exhibits a steady increase in potential over each 5-min current density step. FeCrNi shows a smooth, fluctuation-free rise, while CrCoNi displays slight fluctuations in the potential. These fluctuations may be attributed to CrCoNi’s susceptibility to intergranular corrosion in chloride-containing electrolytes [33]. Although the corrosion mechanism of FeCrNi has not been extensively studied, to the best of our knowledge, the accumulation of bcc phase precipitates at grain boundaries may play a role in mitigating intergranular corrosion [36]. Understanding and integrating such processes into corrosion models could enhance predictive capabilities and inform alloy design strategies which exceeds the scope of this study. However, the CP measurements indicate that alloys susceptible to localized corrosion, such as CrCoNi to intergranular corrosion and CrMnFeCoNi and AISI 304 to pitting corrosion, can be identified by applying constant current densities and by recording the final potential as well as the fluctuations during the polarization.

**Fig. 8:**
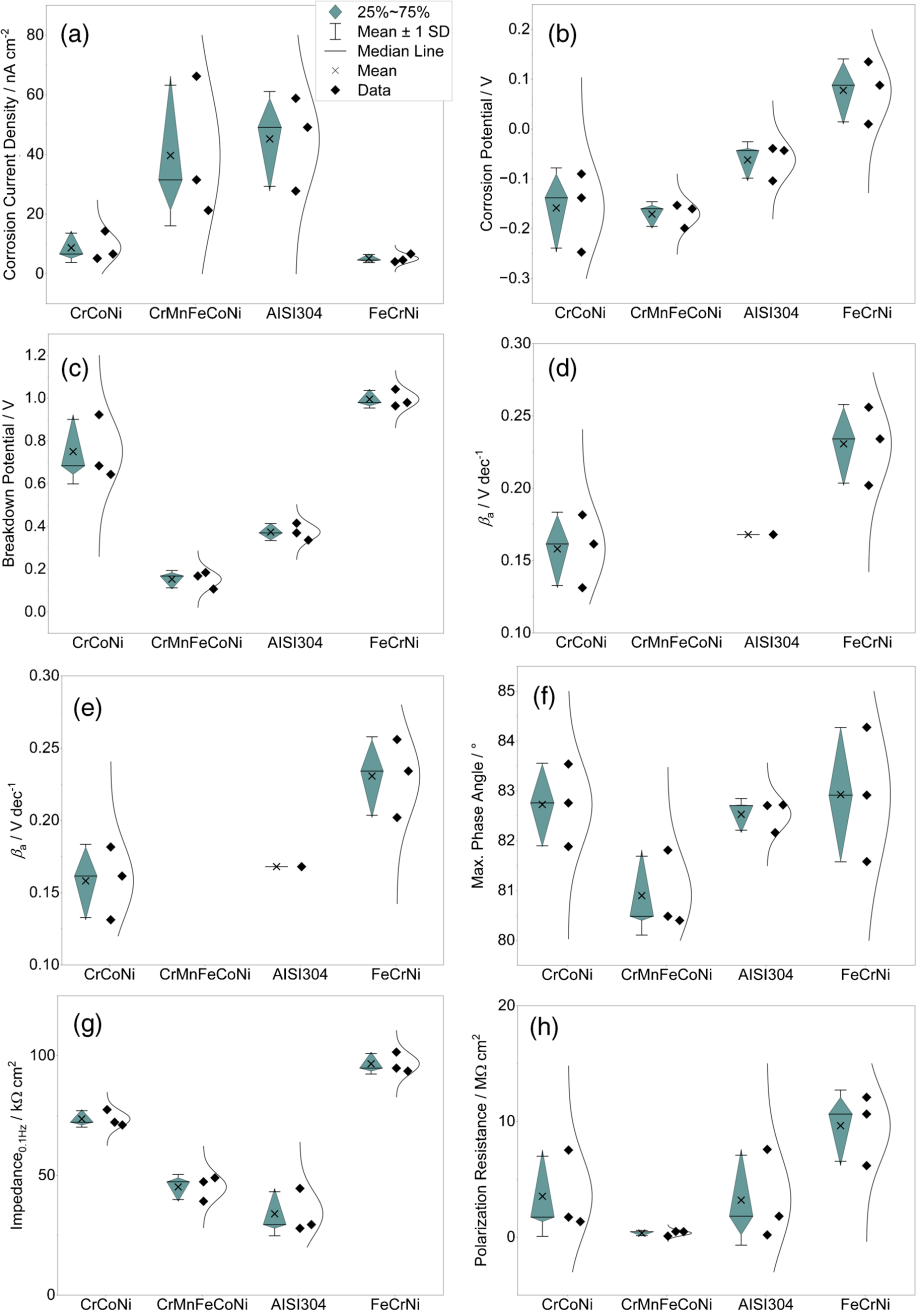
Summary of all electrochemical data from three separate runs. The diagram represent (**a**) *j*_corr_, (**b)** *E*_corr_, (**c)** *E*_b_, (**d)** *b*_a_, (**e)** *b*_c_, (**f)** max phase shift, (**g)** |*Z*|_0.1Hz_, and (**h)** *R*_p_

Figure 8 consolidates all corrosion parameters with their respective standard deviations obtained for each alloy. While corrosion investigations often involve extensive interpretation, the results demonstrate that, when selected and combined carefully, certain parameters can be employed to effectively identify CRAs within a closed-loop MAP. Relying solely on OCP or *E*_corr_ as indicators of nobility would be misleading and could lead to erroneous classifications [52]. Because *E*_corr_ and *E*_b_ show consistency within individual alloys but may vary with environmental conditions, they are better suited for verifying reproducibility of electrochemical runs within the closed-loop rather than directly assessing corrosion resistance. Discerning *E*_corr_ and *j*_corr_ from potentiodynamic scan may not represent the actual values due to interference from capacitive charging. In this study, *E*_corr_ and OCP correlated well for the 4 investigated alloys but more corrosive systems may not replicate this behavior.

Impedance measurements reveal that achieving comparable *R*_p_ values from LPR and EIS requires EIS scans to extend to very low frequencies to be comparable to LPR, particularly for ideally polarizable electrodes. However, this approach increases measurement time and carries the risk of OCP drift and change in surface state, potentially compromising data reliability. Both LPR and EIS offer distinct advantages. LPR is straightforward, easily interpretable, and well-suited for automation, but its accuracy depends on OCP stability and at high corrosive load, the deviations may be too large to be used as a reliable indicator. Furthermore, the distortion between *E*_corr_ and *E*_OCP_ due to capacitive charging during potentiodynamic polarization can be observed for CrCoNi and FeCrNi which in turn also affects the CPP results. EIS data seemed to remain reproducible over the given timeframe despite the temporal changes in OCP. Impedance data may provide highly detailed insights into the interfacial processes of electrochemical systems but its analysis is complex and often requires human supervision, although steps are being taken to autonomously fit EIS data with electrical equivalent circuits through software tools such as AutoEIS [53]. Nevertheless, extracting the |*Z*| at 0.1 Hz and the maximum phase angle can serve as practical indicators, with higher values correlating to greater corrosion resistance and capacitive capability of the passive film. Combining LPR and EIS_0.1Hz_ enables the acquisition of *R*_p_ from LPR and get a ranking from |*Z*| at 0.1 Hz and the maximum phase angle from EIS, offering a more robust evaluation framework.

Since two parameters from the same measurement alone cannot fully characterize corrosion resistance and may carry statistical error, additional metrics such as *j*_corr_ and the cathodic Tafel slope should be included. To avoid erroneous fitting of the anodic slope, only the cathodic Tafel slope should be considered to ensure data stability. Care should, however, be taken when using these corrosion metrics at their face value since the potentiodynamic scan parameters and resulting effects such as capacitive charging affect the results. This emphasizes the importance of metadata to accompany corrosion metrics in respective databases. The results show that a typical electrochemical sequence comprising OCP-EIS_0.1Hz_-LPR-CPP can provide comprehensive data that comprises |*Z|*_0.1Hz_, max phase angle, *R*_p_, *j*_corr_, *E*_corr_, and *E*_b_. Such a sequence would surmount to approximately 1 h 15 min. However, this may leave the sample fully altered due to forced anodic corrosion. Furthermore, autonomously discerning pitting corrosion from the CPP remains challenging. To address this, a quasi-stationary chronopotentiometric step test (lasting 20 min) at increasing current densities was conducted within this study. Requiring no upfront OCP stabilization, the fluctuation in the response potential provides a fast indicator of localized corrosion. Consequently, a sequence comprising OCP-EIS_0.1Hz_-LPR-Tafel-CP could be more time-efficient while generating the informative corrosion metrics that allow to determine the most corrosion-resistant material.

## Conclusions

In this study, we evaluated the feasibility of different corrosion parameters to be employed in a material acceleration platform with the goal to identify a minimal, time-efficient, and informative electrochemical measurement sequence for corrosion resistance evaluation. While there exists a plethora of corrosion parameters, *R*_p_, *j*_corr_, *b*_c_, *E*_corr_, and *E*_b_ were found to be most suitable for uniform corrosion resistance identification. From the reproducibility of EIS data over time, it can be concluded that there is no need to extensively monitor OCP. Distortions between *E*_corr_ and *E*_OCP_ can be identified from the potentiodynamic scans (LPR and CPP). Although the modulus of impedance at 0.1 Hz is suggested to rank different alloy systems for their polarization resistance capability (and hence corrosion resistance), the actual polarization resistance should be retrieved from LPR measurements. *E*_corr_, *j*_corr_, *b*_c_, and *E*_b_ can be extracted from CPP scans. However, even though CPP scans hold a lot of corrosion-relevant information, appropriate algorithms need to be designed that can reliably extract the desired information such localized corrosion.

To enhance sensitivity to localized corrosion phenomena, chronopotentiometry was examined. The temporal evolution of the potential under controlled current density application provided a clear and rapid indicator of localized corrosion susceptibility, enabling the identification of pitting behavior that is otherwise difficult to extract reliably from CPP data alone. Under the conditions studied, chronopotentiometry offered the best combination of diagnostic value and execution time, making it particularly well suited for deployment in self-driving laboratory workflows. Realizing the full potential of this framework will require extensive, metadata-rich electrochemical databases to support autonomous evaluation and model development. High‑throughput electrochemical platforms—such as flow‑cell‑based SDL set-ups—provide a viable route for generating such datasets and accelerating materials discovery in alloy, thin‑film, and catalyst design. Overall, the proposed electrochemical measurement sequences represent a meaningful step towards standardizing accelerated electrochemical characterization and enabling its seamless integration into MAP-driven materials development pipelines.

## Data Availability

All data supporting the results and all scripts used in measuring and evaluating the data will be submitted to Zenodo [38] during/after the revision process. This text will be replaced with the DOI of the dataset.
